# Production, Purification, and Biochemical Characterization of a Novel ATP-Dependent Caseinolytic Protease from the Marine Bacterium *Cobetia amphilecti* KMM 296

**DOI:** 10.3390/microorganisms13020307

**Published:** 2025-01-30

**Authors:** Yulia Noskova, Olga Nedashkovskaya, Larissa Balabanova

**Affiliations:** G.B. Elyakov Pacific Institute of Bioorganic Chemistry, Far Eastern Branch, Russian Academy of Sciences, Prospect 100-Letya Vladivostoka 152, 690022 Vladivostok, Russia; oned2004@mail.ru

**Keywords:** marine bacterium, *Cobetia amphilecti*, ATP-dependent Clp endopeptidase proteolytic subunit ClpP, serine protease, recombinant protein, proteolytic activity

## Abstract

A novel caseinolytic protease (ClpP) of the S14 family from *Cobetia amphilecti* KMM 296 (CamClpP), comprising 206 amino acids, with a calculated molecular weight of 22.66 kDa and a pI of 4.88, was expressed in *Escherichia coli* cells to verify the functional annotation of the encoding gene that has low identity with known structures. The proteolytic activity of the purified recombinant enzyme was found to be 2824 U/mg, using 1% casein as a substrate. Enzyme activity was maximal at pH 5.6 and 7.4 in phosphate buffer and was maintained over a wide pH range of 4-10. The optimum temperature for protease activity was 45 °C. The enzyme in its optimal state required the presence of either NaCl or KCl at concentrations of 0.3 and 0.2 M, respectively. The addition of the metal ions Mg^2+^, Ca^2+^, Ni^2+^, Mn^2+^, Li^+^, and Zn^2+^ at 2 mM resulted in a significant inhibition of the protease activity. However, the presence of Co^2+^ led to a marked activation of the enzyme in the absence of ATP. The enzyme activity was inhibited by ethanol, isopropanol, glycerol, SDS, EGTA, and EDTA. The presence of Triton X-100, acetone, DTT, and PMSF resulted in a significant increase in the CamClpP protease activity. The protease CamClpP effectively and preferentially degrades high-polymer wheat and rye flour proteins. This new proteolytic enzyme with unique properties is of great ecological and biotechnological importance.

## 1. Introduction

ATP-dependent caseinolytic proteases are multi-subunit enzymes which play an important role in controlling protein quality and quantity (proteostasis) by degrading misshapen, damaged, short-lived, or regulatory proteins and are involved in many physiological processes in bacteria and mitochondria [1,2,3,4,5,6,7]. The ATP-binding subunits (ClpX, ClpA, ClpC, or ClpE) form hexamers and interact with two heptameric rings of a self-compartmentalized serine peptidase ClpP (ClpP-ATPase) that are stacked on top of each other, with the catalytic triads Ser-His-Asp facing inward within the proteolytic chamber. The ATPase acts as a molecular chaperone that recognizes and catalyzes the unfolding of stable proteins if they have an open site or certain degradation tags are exposed under certain conditions. Then, the unfolded polypeptide chains are translocated into the proteolytic chamber of homo- or hetero-tetradecameric ClpP for degradation and the following release [1,2,3,4,5,6,7]. А few pathogenic bacteria, such as *Streptomyces* spp., *Chlamydia* spp., *Clostridium* spp., *Listeria* spp., or *Pseudomonas aeruginosa*, encode two or more ClpP isoforms [1,2]. The Clp proteases are known to be involved in various stress responses and the regulation of biofilm formation in many pathogenic bacteria; they are allosterically multilayered and activated by their chaperons, substrates, and active-site inhibitors [4,5,6,7].

According to the MEROPS database (https://www.ebi.ac.uk/merops/, accessed on 21 May 2024), proteases are classified mainly on the basis of their phylogenetic relationships and mechanism of action [8]. Each protease is assigned to a specific family based on statistically significant similarity in amino acid sequence, and families that are considered homologous are grouped into a clan based on indels. The cytosolic ATP-dependent caseinolytic proteases ClpP and their homologues are grouped in the S14 family [8].

The study of Clp proteases is important in biology and medicine because these enzymes play a key role in the regulation of protein metabolism and the removal of damaged or misfolded proteins from the cell. In general, the focus of the research on ClpP can be related to the understanding of the function of these enzymes in the regulation of protein metabolism, disease pathogenesis, and cellular defense, as well as the development of new treatments and therapies [1,2,4,5,6,7,9]. However, very little is known about the structure, function, and role of these enzymes in marine ecosystems. Enzymes isolated from marine bacteria occupy a special place in research because the conditions of life in highly saline, hot, or arctic waters suggest the unique properties of such enzymes, which may lead to the development of new biotechnological applications [10,11,12].

The *Halomonas*-like bacteria of the genus *Cobetia* are found in a variety of locations across the northwest Pacific, including *Cobetia amphilecti* strain KMM 296, which was isolated from a mussel; it was collected in the Sea of Japan and showed growth at a wide range of temperatures (4 °C to 42 °C) and salinity (0.5–20%) [10]. Whole-genome analyses of *Cobetia* spp. isolates have revealed the existence of highly versatile strain-dependent metabolic pathways, biotechnology-relevant biosynthetic gene clusters, and cold-adapted or moderately thermostable enzymes with unknown substrate specificity and biological functions [10,11]. The species *C. amphilecti* was found to possess multiple representatives of the alkaline phosphatase and protease families, indicating a comprehensive adaptability developed across evolutionary time [10,11]. The coding sequences of *C. amphilecti* have low identity with the studied protein structures and are therefore of interest for finding new functions and applications in various fields, including microbiology, biotechnology, medicine, and ecology. For example, a functional annotation for the *C. amphilecti* KMM 296 gene encoding for a DegP (DegQ)-like protease was previously confirmed [11].

The aim of this study was to investigate the recombinant production, purification, proteolytic activity, and physicochemical properties of a novel Clp-like protease, the coding sequence of which was isolated from the genome of the marine bacterium *C. amphilecti* KMM 296 (Collection of Marine Microorganisms, G.B. Elyakov Pacific Institute of Bioorganic Chemistry, Far Eastern Branch of the Russian Academy of Sciences (PIBOC FEB RAS)) and annotated as “ATP-dependent Clp endopeptidase proteolytic subunit ClpP” (GenBank IDs: WP_043331300.1, KGA03297.1).

## 2. Materials and Methods

### 2.1. Materials and Reagents

High-purity reagents from Merck (Munich, Germany), Sigma (Sigma-Aldrich Rus LLC, Moscow, Russia), and Helicon (Moscow, Russia) were used. DNA isolation, restriction, ligation, oligonucleotide, and Taq polymerase kits were purchased from Evrogen (Moscow, Russia) and Thermo Fisher Scientific RU (Moscow, Khimki, Russia); kanamycin was produced by Synthesis (Moscow, Russia). Yeast extract, bactoagar, tryptone, and peptone were purchased from Helicon and Dia-M (Moscow, Russia). DNA and protein molecular weight markers were purchased from BioRad (Bio-Rad Laboratories, Inc., Hercules, CA, USA).

### 2.2. Construction of pET40 CamClpP Plasmid

The recombinant plasmid pET40 CamClpP was constructed by inserting a full-length gene encoding for the Clp-like protease (GenBank IDs: WP_043331300.1, KGA03297.1) into the NcoI/SacI region of the plasmid pET-40b(+) (Thermo Fisher Scientific-RU, Moscow, Khimki, Russia), which was synthesized by polymerase chain reaction (PCR) using the genomic DNA of *C. amphilecti* KMM 296 (KMM, PIBOC FEB RAS, https://kmm644.ru accessed on 14 November 2024) and the following gene-specific primers: CamClpP-NcoI-dir: 5′-TATCCATGGTAAACGACTTCGACATCAAGAATGCT-3′ and CamClpP-SacI-rev: 5′-TATAGAGCTCTCACTCCACGTCGGGACGGCGTTCC-3′. The reaction conditions were as follows: 1 µL of 10× Encyclo buffer, 0.2 µL of 50× Encyclo polymerase mix (Encyclo PCR kit; Eurogen, Moscow, Russia), 0.2 µL of 50× dNTP mix (10 mM each), primer mix (1 µL of 5 µM each), and 1 µL of 20 ng DNA. The volume of the reaction mixture was 10 µL. The amplification process consisted of 38 PCR cycles (15 s, 95 °C; 40 s, 72 °C). After amplification, the PCR product was purified by electrophoresis using a 1% agarose gel. The PCR fragment (1 μg) was treated with the restriction enzymes NcoI and SacI in optimal buffer (Thermo Fisher Scientific RU, Moscow, Khimki, Russia) for 3 h at 37 °C; then, the enzymes were removed from the reaction mixture with phenol (1:1). To the aqueous fraction containing the PCR fragment, 1/10 volume of 0.3 M sodium acetate (pH 5.2) and 1/2 volume of isopropyl alcohol were added and incubated at −20 °C for 30 min. After centrifugation at 14,000 rpm for 20 min, the precipitate was washed with 75% ethanol and dried at room temperature. The precipitate was dissolved in 20 µL deionized water. A total of 2 μg of plasmid DNA pET-40b(+) (Thermo Fisher Scientific-RU, Moscow, Khimki, Russia) was treated with NcoI and SacI restriction endonucleases as described above. The resulting CamClpP gene fragment and the NcoI/SacI part of the pET-40b(+) plasmid were ligated using the ligase reaction in 50 µL ligation buffer according to the instructions (Thermo Fisher Scientific-RU, Moscow, Khimki, Russia). Then, 10 µL of the reaction mixture was used to transform the competent *E. coli* Rosetta (DE3) cells. The transformants were grown on Luria–Bertani (LB) agar containing 50 µg/mL kanamycin. After incubation for 16 h at 37 °C, clones were screened, and the target plasmid DNA was isolated and screened for mutations.

### 2.3. Production of Recombinant Protease CamClpP

The recombinant *E. coli* Rosetta strain (DE3) was grown in 25 mL liquid LB medium containing 50 µg/mL kanamycin at 200 rpm for 18 h at 37 °C. The cells were then transferred to the fresh LB medium (1 L) containing 50 µg/mL kanamycin and incubated at 37 °C on a shaker at 200 rpm until an optical density of 0.6–0.8 at 600 nm was obtained. Then, 0.2 mM isopropyl-β-D-1-thiogalactopyranoside (IPTG) was added to induce the recombinant protein expression. Incubation was continued at 18 °C for 18 h at 200 rpm. The cells were precipitated by centrifugation at 5000 rpm for 15 min at 4 °C, suspended in 20 mL of 50 mM Tris-HCl buffer (pH 8.0), and the cells were disrupted on a Bandeline disintegrator (Germany) at 22 kHz in an ice bath at 40 s intervals until a clear suspension was obtained. The suspension was centrifuged at 11,000 rpm for 20 min at 4 °C, and the precipitate was discarded. The proteolytic activity of CamClpP was determined in the extract obtained, as described below. The protein concentration was determined by the Bradford method [13] using bovine serum albumin (BSA) as a reference standard.

### 2.4. Isolation and Purification of Recombinant Protease CamClpP

The entire isolation procedure was carried out at 4 °C. To isolate CamClpP, dry ammonium sulfate was added to the resulting supernatant, and the protein was fractionated stepwise from 0-40% to 40-60% saturation. The precipitate after 40–60% saturation with ammonium sulphate was dissolved in 50 mM Tris-HCl buffer, pH 8.0 (buffer A), and dialyzed against the same buffer for 16 h. After dialysis, the protein solution was applied to a 25 × 3.2 cm Ni-IMAC-Sepharose column (Cytiva (GE Healthcare) Life Sciences, Marlborough, MA, USA) equilibrated with buffer A. Recombinant protein was eluted with 50 mM Tris-HCl, pH 8.0, containing 0.5 M imidazole and 0.5 M NaCl (buffer B, 10 CV) at 3 mL/min. Fractions containing CamClpP were pooled and purified on a 10 × 1.4 cm Source 15 Q column (Cytiva (GE Healthcare) Life Sciences, Marlborough, MA, USA) equilibrated with buffer A; then, the protein was eluted with a linear gradient of 0-1 M NaCl in buffer A (buffer C, 10 CV). Ion exchange chromatography was performed at 3 mL/min, collecting 1 mL per fraction. Fractions containing CamClpP were pooled and treated with enterokinase at a final concentration of 1 U per 2 mg protein for 18 h at 20 °C. The protein solution was then applied to a HisTrap™ High-Performance column (Cytiva (GE Healthcare) Life Sciences, Marlborough, MA, USA) pre-equilibrated with buffer A. The recombinant protein was eluted with a linear gradient of buffer B at a rate of 0.5 mL/min. Fractions containing CamClpP were collected and concentrated on a Mono-Q HR column (4 × 0.8 cm) (Cytiva (GE Healthcare) Life Sciences, Marlborough, MA, USA) pre-equilibrated with buffer A, washed with 10 volumes of buffer A, and protein was eluted with a linear gradient of buffers A and C at a rate of 0.5 mL/min, collecting 1 mL per fraction. The purified CamClpP preparation was used to study the physicochemical properties and substrate specificity.

### 2.5. Determination of CamClpP Proteolytic Activity

CamClpP protease activity was determined by the method given in [14], using 1% casein as substrate. The CamClpP solution was mixed with 0.5 mL of 50 mM Tris-HCl buffer, pH 8.0, containing 1% casein and 0.5 mM ATP. The mixture was incubated for 20 min at 50 °C. The reaction was stopped by cold 10% trichloroacetic acid (TCA). The acid-soluble products of hydrolysis were determined spectrophotometrically at a wavelength of 280 nm. A standard curve was drawn using tyrosine solutions at concentrations of 0–100 µg/L. One unit (U) of protease activity was defined as the amount of enzyme that releases 1 µg of tyrosine in 1 min under the experimental conditions used. The protease activity is the average of two measurements conducted in triplicate. The difference between the values did not exceed 5%.

### 2.6. Effect of pH on CamClpP Protease Activity and Stability

The determination of the optimum pH for CamClpP activity was studied in the range of pH 3.2–10.0, using 1% casein as a substrate [15]. The following buffer solutions were used in the experiment: 100 mM sodium acetate (pH 3.2–6.2); 100 mM Tris-HCl (pH 6.0–10.0); and 100 mM sodium phosphate buffer (pH 5.0–10.0). Stability at different pH values was tested by incubating the enzyme in 100 mM acetate buffer at pH values of 3.0, 3.5, and 4 and in 100 mM sodium phosphate buffer at pH values of 5.6, 7.4, 9.6, and 10.0, supplemented with 0.5 mM ATP and 0.3 M NaCl for 0, 20, 40, and 60 min; then, 1% casein was added, and the caseinolytic activity was determined as described above.

### 2.7. Determination of Temperature Stability and Optimum Temperature for CamClpP Activity

The effect of temperature on CamClpP activity was determined by incubating the standard incubation mixture at the temperatures ranging from 15 to 65 °C at the intervals 5–10 °C [16]. Temperature stability was determined by the pre-incubation of a solution of CamClpP in 50 mM Tris-HCl buffer (pH 8.0) at different temperatures for 0, 10, 20, 30, 40, 50, and 60 min; then, 1% casein was added to determine the proteolytic activity of the enzyme by the standard assay method, as described above.

### 2.8. Effect of Metal Ions, Chelating Agents, Solvents, and Detergents on CamClpP

The effect of Mg^2+^, Ca^2+^, Mn^2+^, Mn^2+^, Zn^2+^, Co^2+^, Ni^2+^, Cu^2+^, and Li^+^ at the salt concentration of 2 mM and EGTA (ethylene glycol tetra-acetic acid), EDTA (ethylenediaminetetraacetic acid), PMSF (phenylmethane sulfonyl fluoride), DTT (dithiothreitol), Triton-X-100, SDS, ethanol, isopropanol, glycerol, and acetone at various concentrations on CamClpP was determined by the standard proteolytic activity assay against the control incubation mixture without cations, chelating agents, solvents, and detergents. The effect of metal ions was determined in both the presence and absence of 0.5 mM ATP. The effect of solvents and other chemicals was determined in the presence of 0.5 mM ATP.

### 2.9. Effect of NaCl and KCl on CamClpP Activity and Stability

The salts NaCl and KCl were added to the standard incubation mixture at different concentrations ranging from 0 to 3 M to determine the optimum ionic strength for CamClpP proteolytic activity. Salt tolerance was determined by the pre-incubation of CamClpP solution under optimal conditions with different concentrations of NaCl from 0 to 3 M in 20 mM sodium phosphate buffer at pH 7.5 for time intervals of 0, 20, 40, and 60 min; then, the proteolytic activity of the enzyme was measured as described above.

### 2.10. Effect of ATP on CamClpP Activity

The effect of ATP on CamClpP activity was studied by the addition of ATP solution at different concentrations from 0 to 10 mM to the standard incubation mixture. The proteolytic activity of CamClpP was measured as described above.

### 2.11. Determination of CamClpP Molecular Weight

The molecular weight of CamClpP was determined by SDS-PAGE [17] and gel filtration with the use of a calibrated Superdex 200 PG column (105 × 2 cm) (Cytiva (GE Healthcare) Life Sciences, Marlborough, MA, USA). Gel filtration was performed in 50 mM Tris-HCl buffer, pH 8.0, with 0.15 M NaCl at a rate of 0.5 mL/min. A Bio-Rad gel filtration standard mix (Bio-Rad Laboratories, Inc., Hercules, CA, USA) containing thyroglobulin (bovine) 670,000 Da, g-globulin (bovine) 158,000 Da, ovalbumin (chicken) 44,000 Da, myoglobin (horse) 17,000 Da, and vitamin B12 1350 Da was used for calibration according to the manufacturer’s recommendations.

### 2.12. Determination of CamClpP Substrate Specificity

The substrate specificity of the protease was determined using the synthetic substrates α-benzoyl-Arg-p-nitroanilide (BAPNA) and N-succinyl-L-alanyl-L-alanyl-L-prolyl-L-phenylalanine-4-nitroanilide (SAPNA) (Sigma Aldrich, St. Louis, MO, USA) and natural substrates, such as casein and bovine serum albumin (Sigma). The high-polymer substrates, such as maize, rye, wheat, and soya grains, were purchased from a local market.

Immediately prior to the experiment, 25 mM standard solutions of BAPNA and SAPNA were prepared in 5% dimethyl sulfoxide (DMSO). Protease activity was assayed by mixing 0.5 mg purified enzyme with 1mL of the buffer containing the substrate (1 mM BAPNA or 20 mM SAPNA with 50 mM Tris-HCl, pH 7.5). The reaction was stopped by adding 1 mL of cold 10% TCA after incubation at 50 °C for 30 min and then held at room temperature for 15 min. The increase in absorbance at the corresponding wavelength (λ 410 nm) due to the enzymatic hydrolysis and release of peptides or *p*-nitroanilide was measured relative to the initial substrate solution without enzyme treatment. The presence of protease activity in CamClpP was confirmed by the protease activity against casein as a control. The amount of enzyme capable of releasing 1.0 mM *p*-nitroanilide per minute was taken as a unit of activity, the concentration of which was determined using a molar absorption coefficient of 10,500 M^−1^ cm^−1^ at a wavelength of 410 nm [18].

Flour from each cereal (soybean, wheat, rye, and maize) was ground in a household blender; then, a 1% solution of the resulting flour in buffer D (50 mM Tris-HCl, pH 8.0, 0.3 M NaCl, 0.5 mM ATP, 5% ethanol) was prepared and incubated at 37 °C for 60 min with constant stirring. After incubation, the solutions were centrifuged at 10,000 rpm for 5 min, and the optical density of the supernatant was measured. Each sample was then diluted with buffer D to an optical density of approximately 0.5, and CamClpP protease was added. The reaction was run at 45 °C for 60 min and then stopped by the addition of 1 volume of cold 10% TCA. Optical density was measured at λ 280 nm. Buffer D without flour but with the addition of CamClpP enzyme was used as a control.

### 2.13. Nucleotide and Amino Acid Sequence Analysis

The MEROPS database (http://merops.sanger.ac.uk/ accessed on 21 May 2024) was used for the *CamClpP* structural classification and amino acid sequence analysis [8]. Analysis of the *CamClpP* gene homology was carried out using the BLAST program available at the portal of the US National Center for Biotechnology Information (NCBI), using the nucleotide blast algorithm (https://blast.ncbi.nlm.nih.gov/Blast.cgi accessed on 14 November 2024). Phylogenetic analysis was performed using the MEGA 11 (Molecular Evolutionary Genetics Analysis) software package, version 11.0.13 (http://www.megasoftware.net/, accessed on 8 November 2024). Ancestral states were determined using maximum likelihood and the JTT matrix model. The tree used a set of possible amino acids (states) at each ancestral node based on their estimated probability at site 1. For each node, only the most probable state was displayed. The initial trees for the heuristic search were obtained by applying the neighbor-join and BioNJ algorithms to the pairwise distance matrix estimated using the JTT model and then selecting the topology with the best log-likelihood value. Rates between sites were assumed to be uniform (uniform rates option). Seven amino acid sequences were included in the analysis; they were retrieved from the NCBI, UniProt, and MEROPS databases on the basis of a BLAST search using the CamClpP protease as the query sequence. A total of 482 positions were included in the final dataset.

## 3. Results and Discussion

### 3.1. CamClpP Structure Analysis

**Figure 1:**
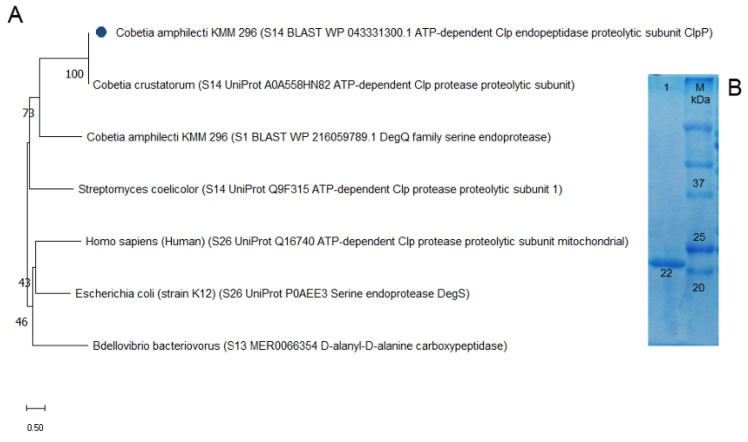
Protein structure analysis and molecular weight of the *C. amphilecti* KMM 296 protease CamClpP: (**A**) Phylogenetic tree based on the analysis of CamClpP protein sequences and closest relatives using maximum likelihood and JTT matrix-based model (MEGA11); (**B**) SDS-PAGE of the purified recombinant protein CamClpP (line 1); M-molecular weight marker (Bio-Rad).

Based on the MEROPS database structural classification, the recombinantly produced protein CamClpP of the marine bacterium *C. amphilecti* KMM 296, encoded by the gene of GenBank ID: WP_043331300.1 (KGA03297.1), belongs to the ATP-dependent proteolytic subunit of the Clp protease [8,19]. According to the results of the Protein Parameters web app (ProtParam, https://protparam.net/, accessed on 28 May 2024), the grand average of hydropathicity and hydrophobicity (GRAVY) and the aliphatic index of CamClpP protease are −0.113 and 97.57, respectively. A GRAVY index below zero indicates that the protein is globular and hydrophilic and dissolves well in aqueous buffer solutions. The high aliphatic index indicates the thermostability of the CamClpP protease. According to the BLAST-based searches, the CamClpP-like protein coding sequences *Cobetia* spp. related to the CamClpP-like protein are close to the biochemically studied ATP-dependent Clp protease proteolytic subunit 1 of the S14 family from *Streptomyces coelicolor* [20], with 51% identity (Figure 1A).

The proteases of the S26 family, which include the ATP-dependent proteolytic subunit of the Clp protease from *Homo sapiens* [21], are about 57.5% identical to those of the *Cobetia* species (Figure 1A). The *E. coli* serine endoprotease DegS [22] and the *Bdellovibrio bacteriovorus* [23] enzyme are not identical to the caseinolytic protease CamClpP (Figure 1A). Thus, the CamClpP-like proteins from *Cobetia* spp. are phylogenetically distant from other proteases, including the S14 family representatives, indicating a new structural member of the ATP-dependent Clp protease of the serine protease families (Figure 1A).

### 3.2. Heterologous Expression and Isolation of CamClpP

The heterologous expression of the *C. amphilecti* KMM 296 gene (GenBank ID: WP_043331300.1, KGA03297.1) corresponding to the mature CamClpP protein in the *E. coli* Rosetta DE3(+) cells resulted in a soluble recombinant CamClpP protein with a specific proteolytic activity of 2824 U/mg (1% casein) after purification according to the scheme described in Table S1.

The mature protease CamClpP contains 206 amino acid residues and has a molecular weight of 22–23 kDa according to the SDS-PAGE electrophoresis data (Figure 1B), which corresponds to the ProtParam calculated value of 22.66 kDa (pI 4.88).

The molecular weights of caseinolytic proteases from marine bacteria have also been reported to range from 21 to 60 kDa [12]. The proteases from *Bacillus* sp. APCMST-CS4, *Yarrowia lipolytica* YlTun15, and *P. aeruginosa* are 21 kDa, 35 kDa, and 60 kDa, respectively [24,25,26]. The molecular weight of caseinolytic protease from *Lactobacillus plantarum* IIA-1AS is 26 kDa [27].

According to the results of gel filtration on a Superdex-200 column, the protease CamClpP from *C. amphilecti* KMM 296 forms a heptameric structure with a peak at around 150 kDa that corresponds to the proteolytically active ClpP structures from other sources [1,2,3,4,5,6,7]. The *clpP* gene is often a single copy, as in *E. coli* or *Bacillus subtilis* [28], but some bacteria, such as *Mycobacterium tuberculosis*, *Chlamydia trachomatis*, *Listeria monocytogenes*, *Clostridioides difficile*, *P. aeruginosa*, and *Leptospira interrogans*, possess multiple *clpP* genes [1,2]. In addition, the ClpP proteins may exist in the form of hetero-oligomeric quaternary structures that are tightly related to their catalytic activity [1,2,29]. The genome of *C. amphilecti* KMM 296 also contains an additional coding sequence related to the ClpP-like protease (GenBank IDs WP_019016718.1, KGA02124.1), the functionality of which still remains to be confirmed.

Across the species, the ClpP monomer has a conserved architecture consisting of an N-terminal axial loop, a central head domain containing the Ser-His-Asp catalytic triad, and an equatorial knob region that mediates the tetradecamer assembly (Figure S1A) [30]. Although the ClpP subunit is related to a serine protease, it does not exhibit protelytic activity unless it forms tetradecamers and assembles with an unfoldase or chaperone ATPase hexamer, but small peptides can be degraded by the heptameric form (Figure S1B) in the absence of ATPases [1,2,3,4,5,6,7,31]. ClpP forms a barrel-shaped tetradecamer consisting of two overlapping heptamers (Figure S1C), which form a central chamber containing 14 identical catalytic sites separated by two protective axial pores. It is one of the most important proteolytic complexes in bacteria [1,2,32].

### 3.3. Effect of ATP on CamClpP Activity

**Figure 2:**
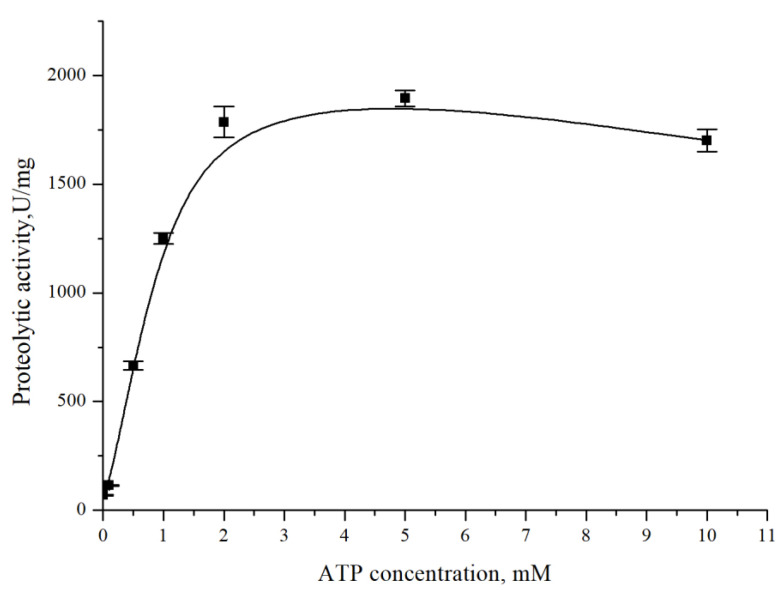
Effect of ATP on the *C. amphilecti* KMM 296 protease CamClpP.

To confirm the dependence of CamClpP on the presence of ATP, adenosine triphosphate was added to the incubation mixture at different concentrations from 0 to 10 mM (Figure 2). The enzyme activity increased sharply at ATP concentrations up to 2 mM, but when the ATP concentration increased from 2 to 10 mM, the protease activity no longer changed.

### 3.4. Effect of pH and Ionic Strength on CamClpP Activity

**Figure 3:**
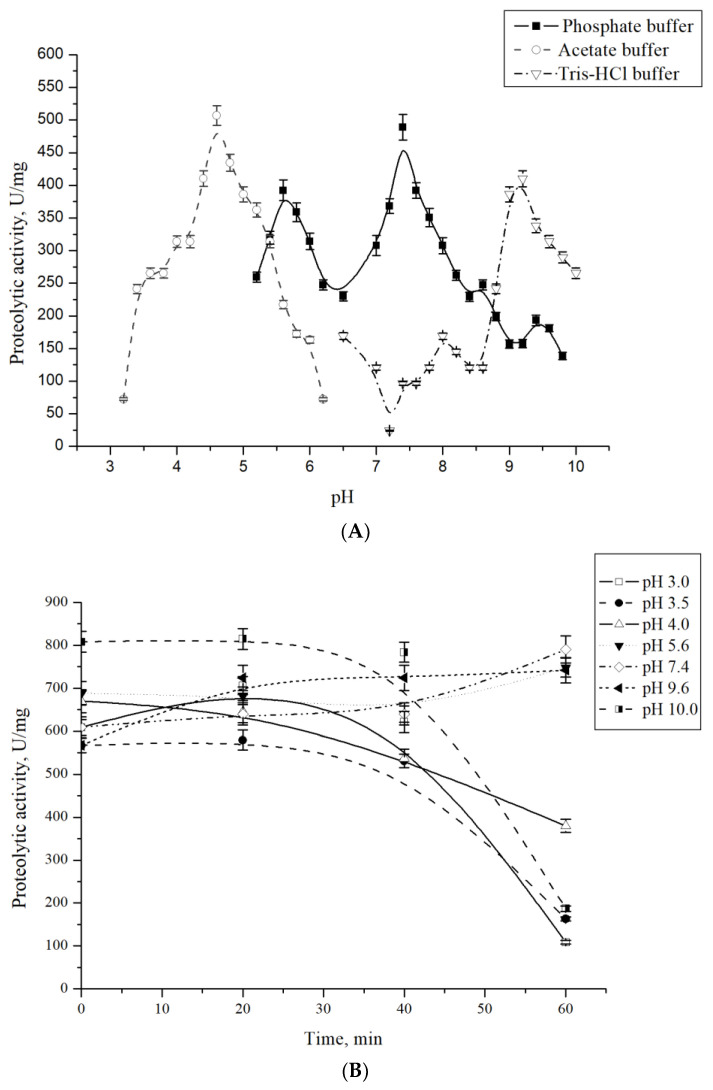
Effect of pH and buffer on the proteolytic activity of *C. amphilecti* KMM 296 protease CamClpP (**A**) and pH stability (**B**).

In nature, ATPases modulate ClpP function by controlling pore access to inhibit promiscuous proteolysis in the intracellular environment, stabilize ClpP in its active state, and select a suitable substrate for degradation [1,2,33]. Changes in intracellular pH control countless biological processes, including ATP synthesis, virus maturation, oligomerization, autophagy, and lysosomal activity, and affect protein structure and function [34,35]. The protease CamClpP of *C. amphilecti* KMM 296 is capable of operating over a wide pH range, but the optimal pH points for its activity are 4.6 in acetate buffer and 9.2 in Tris-HCl buffer (Figure 3A).

In phosphate buffer, CamClpP also showed two peaks of activity at pH 5.6 and 7.4 (Figure 3A). Thus, the enzyme operates in the physiological pH regime but tends to have a burst of activity at extreme pH values. However, CamClpP retained only a third of its activity at pH 3.0–4.0 and pH 10.0 (Figure 3B). Proteases are known to be active over a wide range of pH values, from 4.0 to 11.0. For example, an enzyme isolated from *Vibrio* sp. was active between pH 5.0 and 11.0, with optimum activity at pH 9.0 [36]; a protease from a marine γ-proteobacterium and caseinolytic protease from *L. plantarum* retained the activity between pH 6.0 and 11.0, with optimum of activity at pH 9.0 [27,37]; the results of a study of the serine protease from *Bacillus* sp. APCMST-RS7 also showed a wide pH optimum, pH 6.0–8.0, with maximum values at pH 8.0 [24]. However, some proteases have two optimal pH values. For example, cathepsin B showed the maximum activity at pH 4.6 and 7.2 [38]. The digestive protease of *Centropomus undecimalis* had optimal pH values of 7.0 and 11.0 [39]. Two pH optima can reflect the change in protease conformation in response to pH changes, which is more favorable for substrate binding and catalysis, resulting in elevation in the proteolytic activity. Some proteases can exhibit allosteric behavior, where the binding of a single low-weight molecule affects the activity of the enzyme. If the allosteric effector has a pH-dependent binding affinity, it is possible for the enzyme to have two optimal pH values, each corresponding to a different binding state [35].

**Figure 4:**
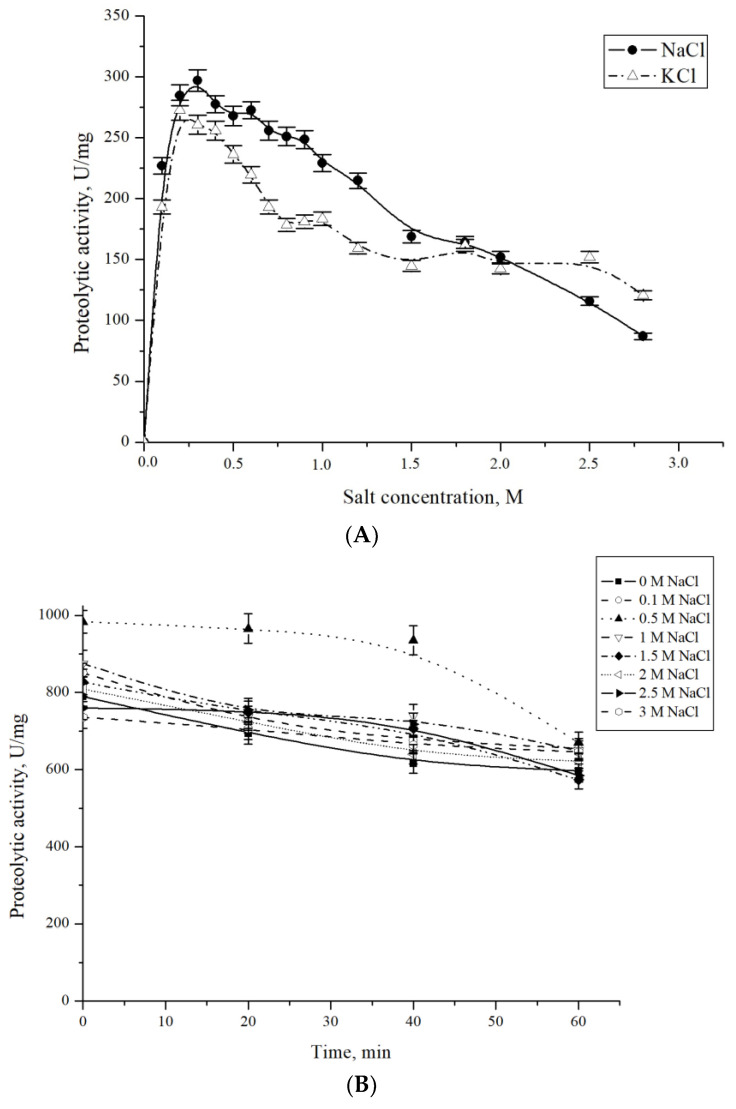
Effect of NaCl and KCl on the *Cobetia amphilecti* KMM 296 protease CamClpP (**A**) and salt stability (**B**).

In addition, the protease CamClpP from *C. amphilecti* KMM 296 was a relatively salt-tolerant enzyme, retaining a third of its activity at NaCl and KCl concentrations up to 2.8 M, with the optimum values at 0.3 M and 0.2 M, respectively (Figure 4A). Halotolerant proteases from marine bacteria of the genus *Bacillus* have been also described as requiring NaCl salt at concentrations of 1.5–2.5 M to function [19,31,32].

As shown in Figure 4B, the CamClpP protease of the marine bacterium *C. amphilecti* KMM 296 is stable when incubated at different concentrations of NaCl, with only a 25% decrease in the activity after one hour of incubation at 3 M of NaCl. To date, several salt-tolerant proteases have been isolated from marine bacteria [12], as well as from salt ponds, saline sediments/saline soils, salt lakes, shrimp paste, deep-sea carbonate rocks, mangrove soil, and sea anemone, which can be used in food and some other industries [40].

### 3.5. Effect of Temperature on CamClpP Activity

**Figure 5:**
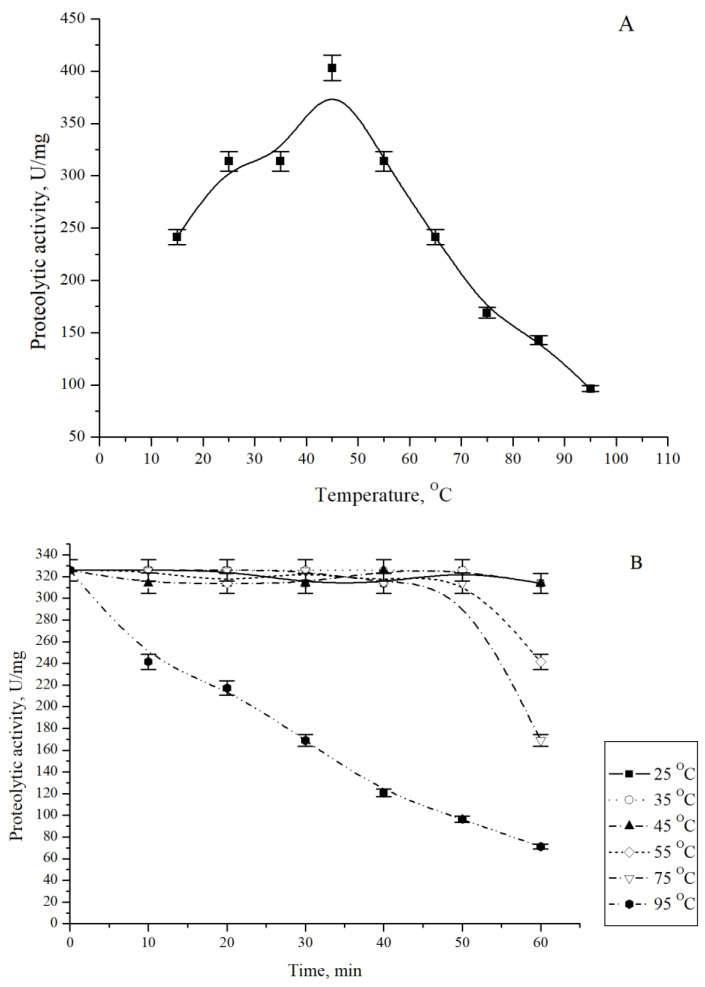
Temperature optimum (**A**) and stability (**B**) of the *C. amphilecti* KMM 296 protease CamClpP.

The optimum of the temperature for the CamClpP proteolytic activity was found to be 45 °C (Figure 5A).

However, the serine protease CamClpP is highly stable, with 50% of enzyme activity retained after 30 min incubation at 95 °C and 30% of activity retained after 50 min incubation at the same temperature (Figure 5B), indicating the capacity of the cold-adapted marine bacterium to retain the viability during the drastic elevation of the environmental temperature. When incubated at 75 °C, the CamClpP protease activity was fully retained for 50 min and decreased by 50% after 1 h incubation at this temperature. The enzyme activity was essentially unchanged when pre-incubated between 25 and 65 °C (Figure 5B). The proteases isolated from marine microorganisms showed optimum temperatures over a wide range, from 40 to 70 °C [12]. For example, the optimum temperatures for the activity of proteases isolated from *Aeromonas* spp., which was collected from wastewater treatment plant in Iran [40], and from the marine yeasts *Aureobasidium pullulans* and *Y. lipolytica* were from 45 to 50 °C [25,41,42], while the optimum temperature for *Bacillus* sp. APCMST-CS4 was 60 °C [24].

### 3.6. Effect of Detergents, Chelators, and Organic Solvents on CamClpP Activity

Table 1 summarizes the effects of protease and metalloprotease inhibitors on the protease CamClpP from *C. amphilecti* KMM 296. EGTA and EDTA are known chelators of metalloproteases. The inhibitory effect of EDTA on CamClpP activity was shown at the concentrations from 2 to 10 mM, whereas EGTA strongly inhibited the enzyme activity even at 2 mM (Table 1).

In contrast, the serine protease inhibitor PMSF activated the CamClpP protease (Table 1). Although PMSF is known as a serine protease inhibitor, it can sometimes stabilize a protease by preventing its denaturation or aggregation. This can increase the activity of the enzyme by maintaining its native conformation and preventing the loss of enzymatic properties. EGTA and EDTA are chelating agents that can affect enzyme activity by binding to a metal ion in the active site and inhibiting activity. Inhibition of the serine protease activity by EGTA and EDTA was observed for enzymes from *Bacillus* sp. AP1 [43], *Pseudomonas lundensis* [44], and *Halobacillus* sp. SCSIO 20089 [45].

As shown in Table 2, ethanol and isopropanol at concentrations up to 5% (*v*/*v*) slightly activated the protease activity of CamClpP, but when their concentrations were increased to 10% (*v*/*v*), they decreased the CamClpP activity twofold with isopropanol and completely inhibited the enzyme when incubated with ethanol.

Inhibition by isopropanol has been observed for proteases isolated from *Geomyces pannorum* and *Chryseobacterium* sp. [46,47]. The addition of glycerol to the incubation mixture reduced the activity of CamClpP protease by four and five times at the concentrations of 5% and 10%, respectively (Table 2).

One of the remarkable features of proteases from marine microorganisms is their stability and activity in the presence of organic solvents. An important reason for the decrease in enzymatic activity in organic solvents is the reduction in the structural flexibility of the enzymes. In aqueous media, enzymes have the conformational flexibility or mobility required for optimal catalysis. Organic solvents, on the other hand, lack the ability to form multiple hydrogen bonds, and they also have lower dielectric constants, resulting in stronger intra-protein electrostatic interactions. As a result, enzyme molecules become more rigid. A major disadvantage of using organic solvents as a medium for enzymatic reactions is that enzymes are easily inactivated or denatured. Therefore, proteases, which are naturally stable in the presence of various organic solvents, are very suitable for synthetic reactions [12]

Triton X-100 and DTT are two chemicals that can significantly increase the enzyme activity (Table 2). Triton X-100 causes conformational changes in the enzyme, making it more active or stable, and can also prevent enzyme aggregation, thereby increasing the concentration of active enzyme [48]. DTT is a reducing agent that can restore disulfide bonds in enzymes, making them more active or stable [12].

The anionic surfactant SDS at a concentration of up to 1% did not affect the activity of CamClpP, but 5% SDS completely inhibited the enzyme. At the same time, 5–10% (*v*/*v*) of acetone and Triton-X-100 increased the CamClpP protease activity manyfold (Table 2); thus, it could have applications in industry and detergent production.

The activity of CamClpP protease increased significantly when DTT was added to the incubation mixture (Table 2). This may indicate that sulphhydryl groups are involved in maintaining the fold or active conformation of the enzyme active site; therefore, the CamClpP protease can be classified as a thiol-dependent serine protease. Similar results were previously obtained for proteases from *Bacillus* sp., *Bacillus subtilis*, *Neocosmospora* sp. N1 [49,50,51,52], and caseinolytic protease from *L. plantarum* [27].

### 3.7. Effect of Metals on CamClpP Activity

To study the effect of metal ions on CamClpP, the caseinolytic activity was measured in the presence of Mg^2+^, Co^2+^, Ca^2+^, Ni^2+^, Mn^2+^, Li^+^, Zn^2+^, and Cu^2+^ salts at a final concentration of 2 mM (Table 3).

Notably, the salt CoCl_2_ showed a twofold increase in the activity of CamClpP, indicating a Co^2+^-dependence similar to that of a number of the recombinant enzymes synthesized from the *C. amphilecti* KMM 296 genes [10,11]. This may indicate a potential contribution of the marine bacterium to the remediation of environments contaminated by cobalt. Copper sulphate had no significant effect on CamClpP activity, whereas the other metal salts studied inhibited the enzyme by either 50% (MgCl_2_, CaCl_2_, ZnCl_2_) or 70% (NiCl_2_, MnCl_2_, LiCl). Some proteases show a dependence on the presence of metal ions in the incubation medium. Most proteases require Mn^2+^, Ca^2+^, and Mg^2+^ ions or their combinations. Metal ions are thought to protect the enzyme from thermal denaturation and to play an important role in maintaining the active conformation of the enzyme at very high temperatures. A protease from *Vibrio* sp. increased the activity in the presence of Ca^2+^, Fe^2+^, and Mn^2+^ cations, whereas Co^2+^, Cu^2+^, Ni^2+^, and Hg^2+^ cations inhibited the enzyme [36]. A protease from the marine bacterium *Proteobacterium proteobacterium* was activated by Ba^2+^ and Ca^2+^ ions, while Co^2+^, Zn^2+^, and Hg^2+^ ions inactivated the enzyme [37]. A multiple increase in protease activity in the presence of cobalt ions was observed for the enzyme from the marine fungus *Geomyces pannorum* [46] and from the bacteria *Aeromonas* spp. [41].

**Figure 6:**
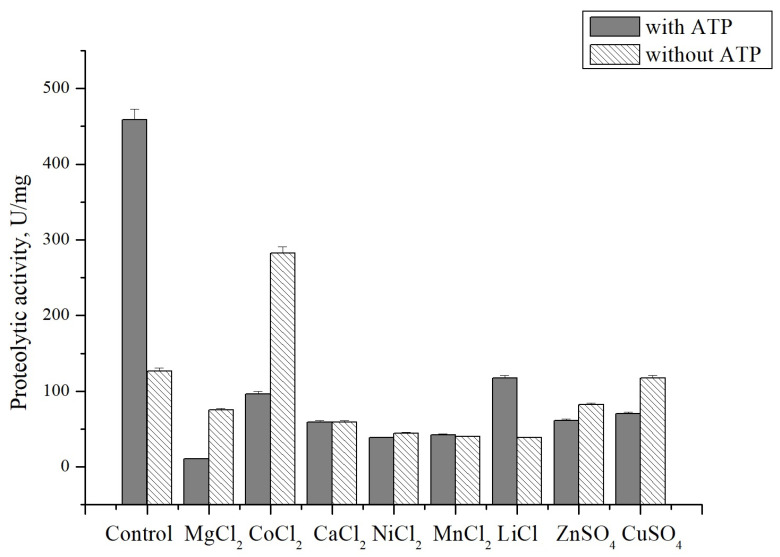
Influence of metal salts on *C. amphilecti* KMM 296 protease CamClpP in the presence and absence of ATP.

It should be noted that the effect of metal salts on the caseinolytic activity of CamClpP was studied in both the presence and absence of ATP in the incubation mixture, since the significant inhibition by the metal ions was observed in the presence of ATP (Figure 6).

However, Co^2+^ had a comparatively lower inhibitory effect on CamClpP, which coincides with the Co^2+^ preference of many enzymes from *C. amphilecti* KMM 296, indicating a strain-specific adaptation feature to a high cobalt concentration in the environment [11,53]. Moreover, cobalt ions activated the enzyme in the absence of ATP (Figure 6). ATP and bivalent metal ions probably concurrently bind the same allosteric regulation site of CamClpP. The low-molecular-weight inhibitors of the Clp protease active site have been found to drastically up-regulate the activity of these allosteric enzymes [5,6,7].

### 3.8. Substrate Specificity of CamClpP

**Figure 7:**
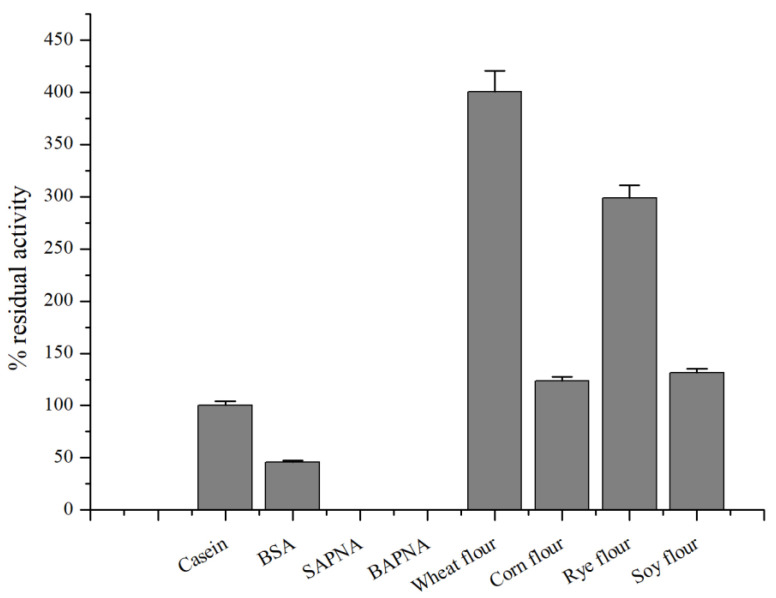
Relative proteolytic activity of *C. amphilecti* KMM 296 protease CamClpP towards proteins of different origins.

The enzyme ClpP is required for the cleavage of peptides in various proteins in a process requiring ATP hydrolysis and has chymotrypsin-like activity [54]. The protease CamClpP of *C. amphilecti* KMM 296 catalyzed the hydrolysis of natural substrates, such as casein, BSA, rye, wheat, soybean, and maize meal (Figure 7).

As shown in Figure 7, the proteolytic activity of CamClpP is similar towards casein, maize meal, and soybean meal, whereas the rates of the enzymatic hydrolysis of rye and wheat flours are 3-fold and 4-fold higher. Although ClpP proteases mostly cleave short peptides [1,2], the common chromogenic substrates for the serine proteases SAPNA and BAPNA are not degraded in the presence of the enzyme CamClpP (Figure 7). A protease that efficiently breaks down proteins from wheat flour has previously been described in the bacterium *C. amphilecti* KMM 296 [11]; furthermore, proteases from various bacteria that break down grain proteins have been described and are widely used by the food and pharmaceutical industries [55,56,57].

## 4. Conclusions

As a result of this study, the gene of the marine bacterium *C. amphilecti* KMM296 with GenBank accession number WP_043331300.1 (KGA03297.1) expressed in *E. coli* cells was shown to encode a metabolically active protease belonging to a structurally novel representative of the ATP-dependent caseinolytic ClpP proteases of the S14 family. The *C. amphilecti* KMM296 CamClpP protease is thermostable, salt-stable, and solvent-resistant and is activated by Co^2+^ in the absence of ATP. The proteolytic enzyme is heptameric under the conditions used (50 mM Tris-HCl buffer, pH 8.0). CamClpP was observed to preferentially cleave the highly polymeric proteins of wheat and rye.

Studies of caseinolytic proteases from marine bacteria, particularly those from *C. amphilecti*, are important and relevant in the fields of microbiology and biotechnology and may have potential applications in various fields, including biotechnology, medicine, and ecology. For example, Clp proteases can be used to develop new methods for protein purification and efficient protein waste recycling systems. These proteases can be used to create new drugs, such as antibacterial or anti-inflammatory agents, due to their ability to cleave certain proteins. Caseinolytic proteases can be used to improve the quality and safety of food products, such as by purifying protein ingredients or removing allergenic proteins. These proteases can be used to develop new diagnostic methods, such as tests for the presence of certain proteins in the body. Clp-like proteases can be used to clean up contaminated water and soil by breaking down protein contaminants.

## Figures and Tables

**Table 1:** Effect of different inhibitors on the proteolytic activity of CamClpP.

Inhibitor	Concentration, mM	% Residual Activity
None	-	100
PMSF	2 5 10	60 115 177
EGTA	2 5 10	0 0 0
EDTA	2 5 10	69 7 0

**Table 2:** The effect of different organic solvents, surfactants, and denaturants on the proteolytic activity of CamClpP.

Reagents	Concentration	Proteolytic Activity, U/mg
None	-	132.7
Ethanol	5% 10%	129.3 0
Isopropanol	5% 10%	156.9 72.4
Glycerol	5% 10%	36.2 24.1
Acetone	5% 10%	12,841.4 15,303.4
SDS	1% 5%	122.4 0
Triton-X-100	1% 5% 10%	8448.3 20,324.1 23,341.4
DTT	2 mM 5 mM 10 mM	2691.4 5901.7 10,331.0

**Table 3:** Effect of metal salts on the proteolytic activity of CamClpP in the absence of ATP in the reaction mixture.

Metal Salts, 2 mM	% Residual Activity
None	100
MgCl_2_	59.2
CoCl_2_	223.0
CaCl_2_	46.6
NiCl_2_	35.2
MnCl_2_	31.4
LiCl	30.5
ZnCl_2_	65.0
CuSO_4_	92.4

## Data Availability

The datasets presented in this study can be found in online repositories. The names of the repository/repositories and accession number(s) can be found in the article/Supplementary Materials: Table S1: Purification scheme of the recombinant *C. amphilecti* KMM 296 protease CamClpP, Figure S1: Three-dimensional model of *C. amphilecti* KMM 296 protease CamClpP generated by AlphaFold software.

## Supplementary Materials

The following supporting information can be downloaded at: https://www.mdpi.com/article/10.3390/microorganisms13020307/s1, Table S1: Purification scheme of the recombinant C. amphilecti KMM 296 protease CamClpP, Figure S1: Three-dimensional model of C. amphilecti KMM 296 protease CamClpP generated by AlphaFold software.
