# Supplementary material for: Production, Purification, and Biochemical Characterization of a Novel ATP-Dependent Caseinolytic Protease from the Marine Bacterium Cobetia amphilecti KMM 296
Source: Microorganisms. 2025 Jan 30;13(2):307. doi: 10.3390/microorganisms13020307 (PMC11857851; doi:10.3390/microorganisms13020307)
Supplement: Supplementary file 1 [file microorganisms-13-00307-s001.zip › microorganisms-3344700-supplementary.pdf]

Table S1. Purification scheme of the recombinant *C. amphilecti* KMM 296 protease CamClpP

Purification step	Total proteolytic activity (units)	Total protein (mg)	Proteolytic activity (U/mg)	Yield (%)	Purification (fold)
Crude homogenate	471128.3	294.4	1600.3	100	1
Ammonium sulfate (40-60 %)	46872.3	43.0	1089.8	10	0.7
Dialysis	21271.1	20.4	1042.7	4.5	0.65
Ni-IMAC-Sepharose	7632.2	23.6	323.4	1.6	0.2
MonoQ	11776.7	4.17	2824.14	2.5	1.8

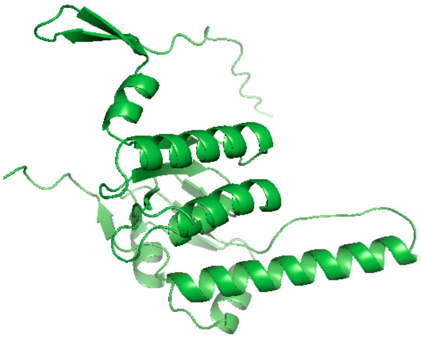

A1

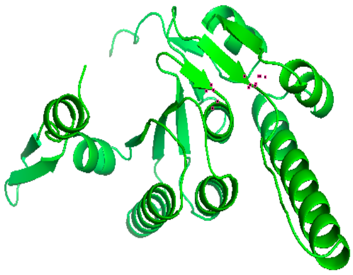

A2

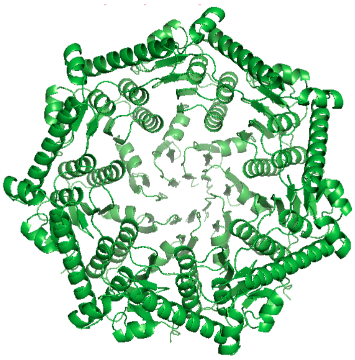

B1

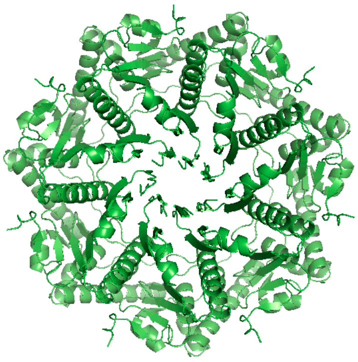

B2

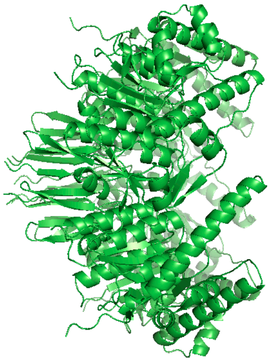

B3

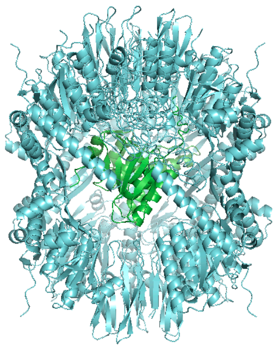

C1

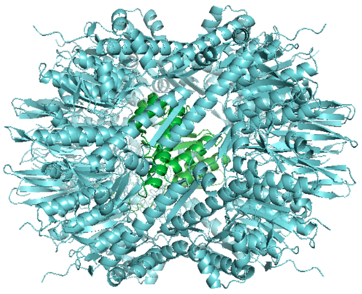

C2

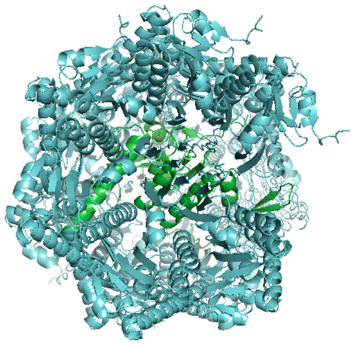

C3

Figure S1. 3-D model of *C. amphilecti* KMM 296 protease CamClpP generated by AlphaFold software. A1 and A2 – monomeric structure of the CamClpP subunit, the active centre of the enzyme is marked in red (A2). B1 - heptamer of CamClpP subunit, bottom view, B2 - heptamer of CamClpP subunit, top view, B3 - heptamer of CamClpP subunit, side view. C1 - dodecamer of CamClpP subunit, side pore shown, C2 and C3 - dodecamer of CamClpP subunit, side and top view. For the monomer, pTM index = 0.92 (no ipTM index). For the heptamer, indices ipTM = 0.86 pTM = 0.88. For the dodecamer the indices are ipTM = 0.63 and pTM = 0.65. The template used was the proteolytic subunit of the ATP-dependent Clp protease from *Cobetia* sp. UCD-24C (UniPro: AF-A0A0P7E240-F1-v4) with 100% amino acid sequence identity to *C. amphilecti* KMM 296. Modelling was performed in AlphaFold software (https://alphafoldserver.com), all structural figures were generated using PyMOL. pTM and ipTM scores: the predicted template modeling (pTM) score and the interface predicted template modeling (ipTM) score are both derived from a measure called the template modeling (TM) score. This measures the accuracy of the entire structure. A pTM score above 0.5 means the overall predicted fold for the complex might be similar to the true structure. ipTM measures the accuracy of the predicted relative positions of the subunits within the complex. Values higher than 0.8 represent confident high-quality predictions, while values below 0.6 suggest likely a failed prediction. ipTM values between 0.6 and 0.8 are a gray zone where predictions could be correct or incorrect. TM score is very strict for small structures or short chains, so pTM assigns values less than 0.05 when fewer than 20 tokens are involved; for these cases PAE or pLDDT may be more indicative of prediction quality.
